# BET, thermal degradation, and FTIR spectras of triazine polyamine polymers

**DOI:** 10.1016/j.dib.2017.02.004

**Published:** 2017-02-16

**Authors:** Mustafa Can

**Affiliations:** Vocational School of Arifiye, Sakarya University, Sakarya, Turkey

**Keywords:** BET, TGA, FTIR, Adsorption

## Abstract

Here we show effect of the polyamine polymer chain length to BET isotherms. According to IUPAC classification [1], all three polymers are fitting type 1 physical adsorption isotherm with H3 hysteresis (except for EDA having H2 hysteresis). Moreover, TG and TGA analysis of polymers triazine-ethylenediamine (EDA) and triazine-triethylenetetramine (TETA) are provided. Due to the similarities of the structure, main decomposition temperatures are close to each other (between 593 K and 873 K). In order to understand change of FTIR spectra with adsorption and stripping Au(III), fresh, Au(III) adsorbed and recycled spectras of polymers measured. For further discussions about the effect of chain length to adsorption of Au(III) onto triazine polyamine polymer particles “Au (III) Uptake by Triazine Polyamine Polymers: Mechanism, Kinetic and Equilibrium Studies” Can et al. [2] (article in press).

**Specifications Table**

**Table:** 

Subject area	*Chemistry*
More specific subject area	*Polymer adsorbent*
Type of data	*Table, image (x-ray, microscopy, etc.), text file, graph, figure*
How data was acquired	The thermal stability behavior of the polymers was carried out by heating from 298 K (25 ^ο^C) to 1073 K (800 ^ο^C) at 40/10 K/min in nitrogen atmosphere by using NETZSCH -STA 449F1 with thermocouples in aluminum pot after milling of the polymers (51.47 mg).
	BET surface area and total pore volume measurements were determined by Gemini 2390 VII using 0.45 g sample.
	FTIR measurements were carried out by Spectrum Two model of Perkin Elmer FTIR spectrophotometer at 4 cm^−1^ resolution in ATR mode using a ceramic light source, KBr/Ge beam splitter, and a LiTaO_3_ detector. The spectra of cyanuric chloride, EDA, TETA, PEHA polymers and Au(III) adsorbed EDA, TETA, PEHA polymers were scanned between 600 and 4000 cm^−1^ for four times.
Data format	*Analyzed,*
Experimental factors	*Amine chain lengths of polymers are changed.*
Experimental features	*The relationship between surface area and polymer amine change length were determined. Change FTIR spectra before, after and recycled polymer as Au(III) adsorbent.*
Data source location	Data are available with the article.
Data accessibility	*The data are available with this article and*http://dx.doi.org/10.1016/j.reactfunctpolym.2016.10.009

**Value of the data**

• Three similar structured polyamine polymers were synthesized for finding best performed Au(III) recycling adsorbent in liquors obtaining from spend electronic devices.
• Information of this data article including, isotherm and kinetic parameters will be informative for modeling and predicting the adsorption capacity and mechanism of Au(III) adsorption onto polymer surfaces.
• This data set will be beneficial for researchers who want to achieve high Au(III) adsorption capacities with N functional group containing polymer adsorbents.

## 1 Data

**Fig. 1:**
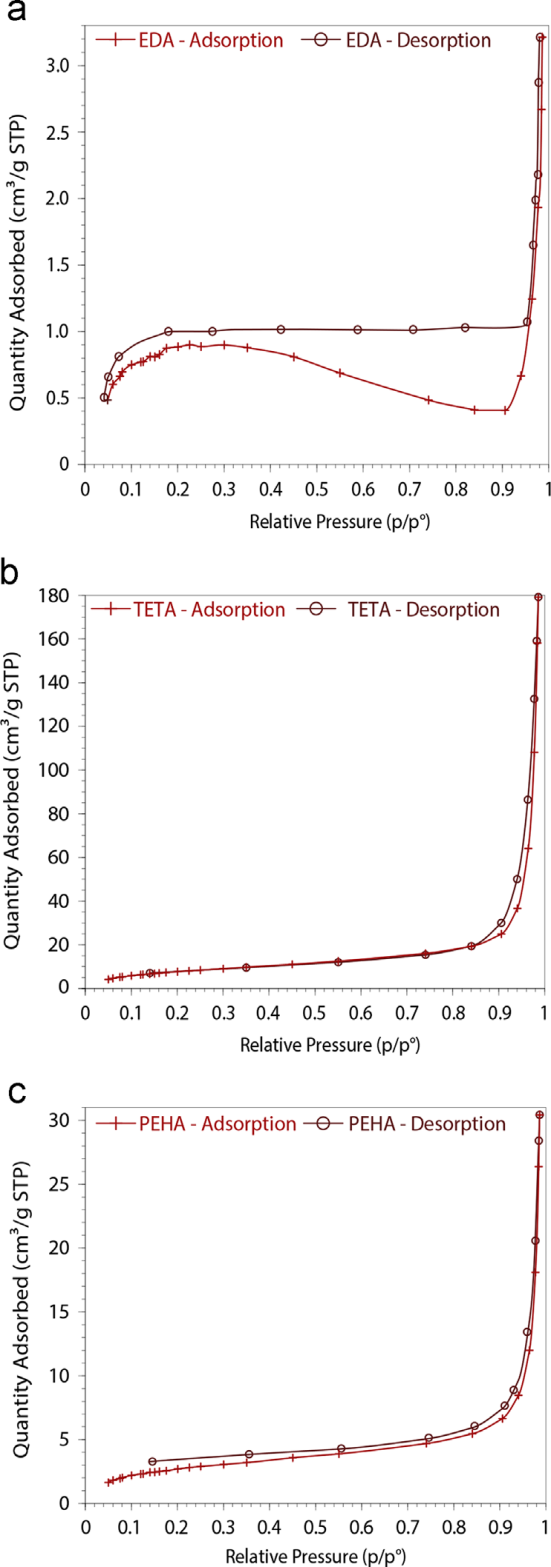
Nitrogen adsorption-desorption isotherms of EDA (a), TETA (b) and PEHA (b) polymers.

**Fig. 2:**
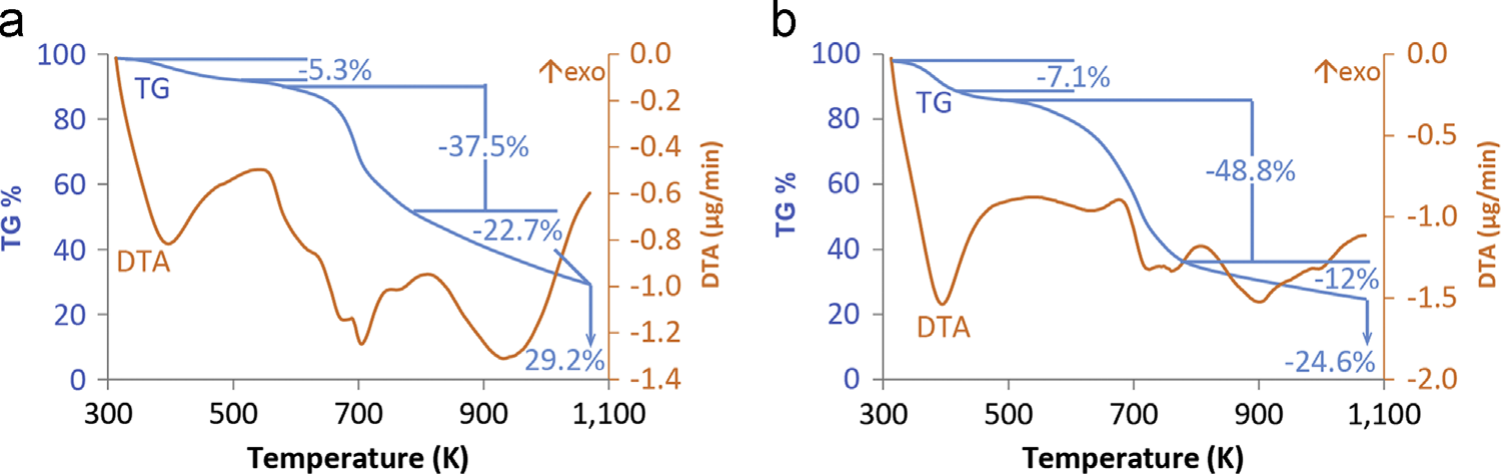
TG and DTG curves of EDA (a) and TETA (b) polymers.

**Fig. 3:**
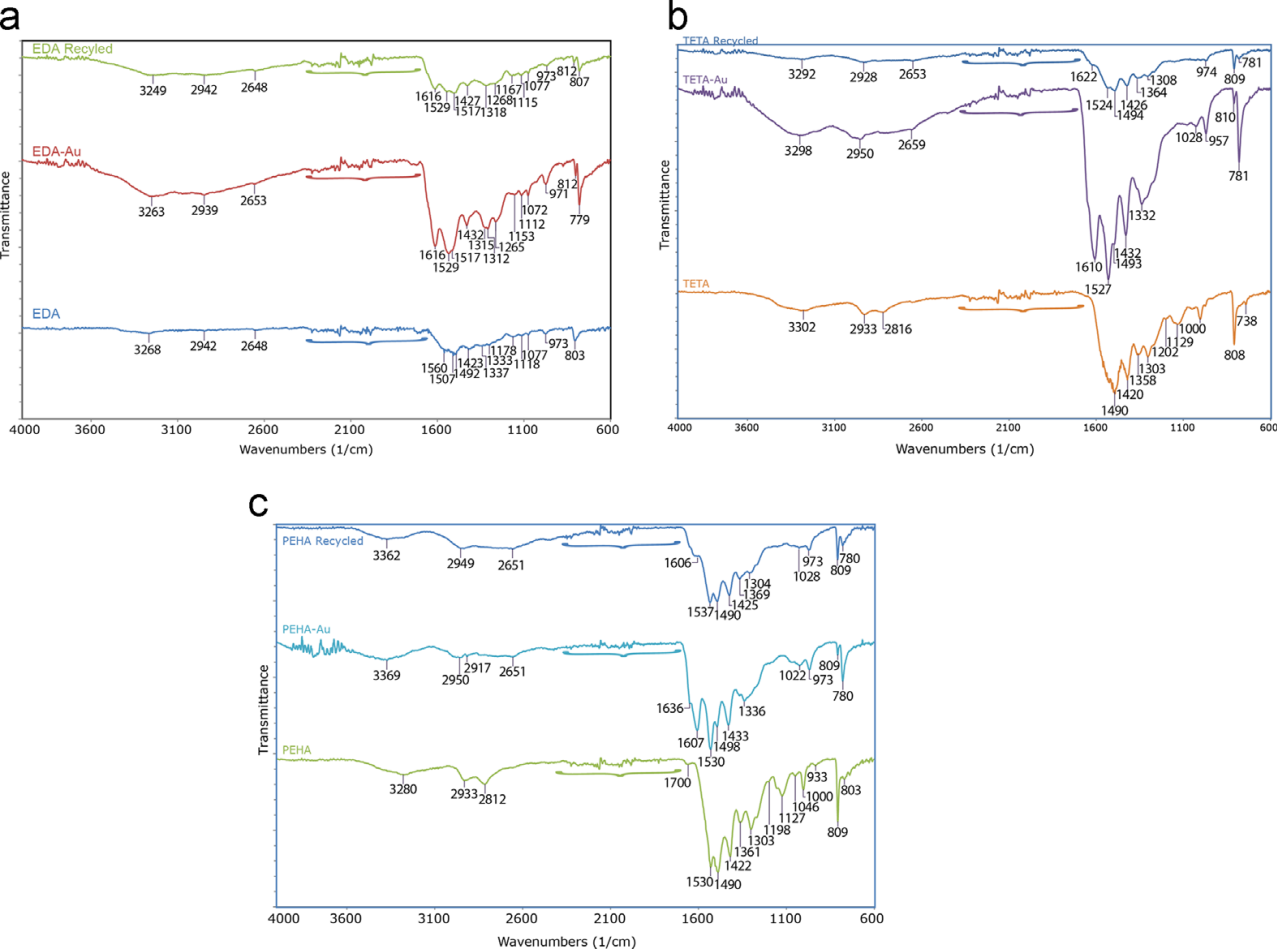
FTIR spectras of EDA (a), TETA (b) and PEHA (c) polymers in fresh, Au-loaded and used several time.

Here, we provided BET isotherms of EDA, TETA and PEHA polymers in Fig. 1. *TG, TGA graphics of EDA and TETA polymer are provided in* Fig. 2. The FTIR of the fresh, Au(III) adsorbed and stripped EDA, TETA and PEHA particles at wave numbers from 600 to 4000 cm^−1^ are shown in Fig. 3.

## 2 BET isotherms

See Fig. 1.

## 3 Thermal degradation results

See Fig. 2.

## 4 FTIR spectras of polymer

See Fig. 3 .

## 5 Experimental design, materials and methods

FTIR measurements were carried out by Spectrum Two model of Perkin Elmer FTIR spectrophotometer at 4 cm^-1^ resolution in ATR mode using a ceramic light source, KBr/Ge beam splitter, and a LiTaO_3_ detector. The spectra of cyanuric chloride, EDA, TETA, PEHA polymers and Au(III) adsorbed EDA, TETA, PEHA polymers were scanned between 600 and 4000 cm^−1^ for four times. The thermal stability behavior of the polymers was carried out by heating from 298 K (25 ^ο^C) to 1073 K (800 ^ο^C) at 40/10 K/min in nitrogen atmosphere by using NETZSCH -STA 449F1 with thermocouples in aluminum pot after milling of the polymers (51.47 mg). BET surface area and total pore volume measurements were determined by Gemini 2390 VII using 0.45 g sample.

In order to optimization of the effecting factors to the Au(III) adsorption, 10 mg of EDA, TETA, PEHA polymers was used. The pH and pCl of Au(III) solutions (25 and 50 mg/L concentration) was adjusted to desired value and batch adsorption experiments were carried out by constant stirring rate using mechanical stirrer or orbital shaker. While only kinetic experiments were carried out by 1000 mL of the solution, all the others were studied using 50 mL. All samples taken for measurements were centrifuged and filtered. Au(III) concentrations in the filtered solutions were measured using Shimadzu 6711F flame atomic adsorption spectrometer (FAAS) . Before measurements, FAAS was calibrated by using 0, 4, 8, 12, 16 and 20 mg/L Au (III) standard solutions and then, the samples were analyzed. Au(III) measurements were carried out by using air-acetylene flame at 242.8 nm and 0.5 nm of slit width. The amount of Au(III) adsorbed on polymers (*q*_e_) was calculated by mass balance as follows:

$$q_{e}=\left(C_{0}-C_{e}\right)V/W$$

where qe is the equilibrium sorption capacity in mg/g; V the volume (L) of the solution; w the weight (g) of the polymer; C_e_ and C_0_ are the equilibrium and initial concentrations of Au(III) (mg/L), respectively. All experiments were performed twice.
